# Medical care provision at the venue of the weightlifting event of the Tokyo 2020 Olympic Games

**DOI:** 10.1007/s11332-021-00865-1

**Published:** 2021-11-19

**Authors:** Kohei Ashikaga, Kihei Yoneyama, Kuniaki Hirayama, Tatsuhiro Suzuki, Ryota Muroi, Rumiko Inoue, Yuki Ishibashi, Junsuke Nakase, Hideaki Takeda, Hiroto Fujiya

**Affiliations:** 1 Department of Sports Medicine, St. Marianna University School of Medicine, 2-16-1 Sugao, Miyakaeku, Kawasaki, Kanagawa 216-8511 Japan; 2 Division of Cardiology, Department of Internal Medicine, St. Marianna University School of Medicine, Kawasaki, Kanagawa Japan; 3 Faculty of Sport Sciences, Waseda University, Saitama, Japan; 4 Department of Rehabilitation, MSMC Midori Clinic, Tsu, Mie Japan; 5 Department of Orthopaedic Surgery, Matsuura Orthopaedic Clinic, Tokyo, Japan; 6 Department of Orthopaedic Surgery, Graduate School of Medical Sciences, Kanazawa University, Kanazawa, Japan; 7 Department of Orthopaedic Surgery, Teikyo Orthopaedic Clinic, Tokyo, Japan

**Keywords:** Weightlifting, Olympic Games, Competition, Athletes

## Abstract

**Purpose:**

This study aimed to investigate the medical care provided at the venue of the weightlifting event of the Tokyo 2020 Olympic Games.

**Methods:**

We retrospectively evaluated athletes who availed of medical services at the venue during the weightlifting event of the Tokyo 2020 Olympic Games. In total, 194 athletes participated in the weightlifting competition.

Injuries and illnesses were classified into cases handled without physician or cases requiring medical examination by a physician. These were tabulated for each examination location (athlete medical station, field of play [FOP], first-aid station of training floor or warm-up area).

**Results:**

Throughout the event, a cumulative total of 132 people used the venue medical services. Nine athletes required medical attention at the athlete medical stations. Of these nine cases, six occurred on the training floor. In the FOP, nine athletes were examined. Two experienced light-headedness, two had knee pain, and the rest had other symptoms. No athletes wanted to undergo medical examination at the athlete medical station. 89 medical procedures were performed in the first-aid station on the training floor, including 52 tapings and 37 ice compressions, and the most frequent body part which needed treatment was the knee, followed by the fingers. At the first-aid station on the match floor, 25 medical procedures were performed, including 17 ice compressions, 5 haemostasis, and 4 tapings. The body parts that most frequently needed treatment were the fingers, knees, and lower legs.

**Conclusion:**

Several athletes needed treatment; however, only a few required medical care from a physician.

## Introduction

The world’s largest sporting event, the Olympic Games, is supported by a variety of industries. An example of this is the medical services industry. The medical system supervised by the Tokyo Organising Committee of the Olympic Games is divided into the medical care from polyclinic at the Olympic village and medical care at each competition venue. The medical care in each competition venue is carried out by the medical teams of the International Federation (IF) and the host country. Injuries and illnesses in the Olympic Games were reported during the entire competition and for each sport or each position [1–4]. This report clarified the occurrence of injuries and illnesses according to the types of sport disciplines.

However, this report did not describe all the medical practices performed by medical staff at each competition venue. This is because such reports only summarize the cases that have been examined by a doctor [1–4]. However, it is not only doctors who provided medical care at the venue but also nurses, physical therapists, and athletic trainers [5–7]. There is no report describing the overall medical practice at one venue, including the medical practices they performed. Therefore, it was unclear what kind of preparation should be made when medical staff other than doctors provide medical care at large-scale sporting events. Since we had the opportunity to provide venue medical care for the weightlifting competition of the 2020 Tokyo Olympics, we thought that clarifying the medical care at the whole venue would support holding large-scale sporting events in the future.

For this reason, this study aimed to investigate the medical care provision in the weightlifting event venue of the Tokyo 2020 Olympic Games.

## Methods

### Weightlifting competition at the 2020 Tokyo Olympics

The weightlifting competition was held in 7 classes for men and women, for a total of 14 classes. The number of participants in each class was 14 without men’s 96 kg class. Only for the men’s 96 kg class, 1 refugee Olympic athlete team participated, so a total of 197 people entered. Each class was permitted only one athlete from each country.

### Study population

We retrospectively evaluated athletes who availed themselves for medical services in the weightlifting event venue of the Tokyo 2020 Olympic Games. The weightlifting event was held at the Tokyo International Forum, which opened between July 19 and August 4, 2021. The medical staff provided medical care to the athletes, International Olympic Committee (IOC) officers, and IF officers. There were about 100 IOC officers and about 140 IF officers. In addition, a total of 194 athletes participated in the weightlifting event of the Olympic Games, excluding 3 who were unable to participate in the match due to injury or SARS-CoV-2 infection.

### First-aid and athlete medical station

In the Tokyo 2020 weightlifting competition, the training and match venues were set up on different floors of the same venue. At the weightlifting venue, one first-aid station was opened on the training floor and two on the warm-up area of the match floor. Each first aid station had at least one athletic trainer, physical therapist, or nurse. Moreover, athlete medical stations were opened on the training and match floor. Each athlete’s medical station had at least one doctor and one physical therapist. These medical services are supported by the host country. There was one IF physician stationed at the warm-up area. During the competition, at least one IF physician, one physician from the host country, and one athletic trainer or physical therapist from the host country stayed in the field of play (FOP).

### Injury and illnesses

Injuries and illnesses were classified as either cases handled by an athletic trainer, physical therapist, or nurse or cases requiring medical examination by a physician. For cases examined only by athletic trainers, physical therapists, or nurses, the details were recorded when the staff directly performed the procedure. Symptoms were recorded for cases examined by a physician. These were tabulated for each examination location (first-aid station of the training floor or warm-up area, FOP, athlete medical station). The number of athletes examined was calculated based on the total number of athletes that required medical attention.

### Medical data collection

A notebook was prepared for each station where the medical staff were assigned, and each station recorded the date and the patient’s gender and country. In addition to this, position and treatment details were recorded at the first aid station. At the medical station or FOP, age, chief complaint, and whether the patient was introduced to the support organization were recorded.

### Ethical approval

This study was performed following the ethical principles set forth in the Declaration of Helsinki, and was approved by the Human Investigation Committee of St. Marianna University School of Medicine (study protocol no. 5396). Informed consent was obtained from the website of the St. Marianna University School of Medicine.

### Statistical analysis

Statistical analysis was performed using the Excel 2019 software (Microsoft Co., Cary, WA, USA). Values are presented as numbers and ratios in percentages. Continuous variables are expressed as mean ± standard deviation.

## Results

During the weightlifting event of the Tokyo 2020 Olympic Games, a cumulative total of 132 people used the medical services in the venue. Of these, 68 (51.5%) were women (Table 1). Additionally, all users were athletes, except for one, who visited the athlete medical station for chest pain.

**Table 1:** Patients at each station

Places	Cumulative total number of people	Gender (female)	Age
Athlete medical station	9	5	31.6 ± 12.3
Field of play	9	3	27.6 ± 3.6
First aid station on the training floor	89	51	Unrated
First aid station on the match floor	25	9	Unrated

A total of 18 cases were examined by the doctors. Their average age was 29.7 ± 9.3 years, with eight women (Table 1). There were nine athletes who needed medical attention at the two athlete medical stations (Table 2). Of these nine athletes, five were female. Of these nine cases, and six of the cases occurred on the training floor. One person, an IF officer, who had chest pain and dyspnoea, was transported to a designated hospital to rule out circulatory or respiratory emergency disease. In the FOP, nine athletes were examined by the competition’s physicians (Table 3), and three of them were female. Two out of the nine cases had light-headedness, knee pain and finger bleeding in two each, and the rest had other symptoms. No athletes wanted to undergo a medical examination at the athlete medical station.

**Table 2:** Symptoms of athletes who consulted at the athlete medical station

Symptom	Number of athletes	Ratio (%)	At the training floor (number)
Headache	2	22.2	2
Diarrhoea	1	11.1	0
Chest pain & dyspnoea	1	11.1	0
Panic attack	1	11.1	0
Lower leg abrasion	1	11.1	1
Ankle pain	1	11.1	1
Thigh pain	1	11.1	1
Nose-wing inflammation	1	11.1	1

**Table 3:** Symptoms of athletes who consulted at the medical station on the field of play

Symptom	Number of athletes	Ratio (%)
Light-headedness	2	25.0
Knee pain	2	25.0
Elbow pain	1	12.5
Hip pain	1	12.5
Shin pain	1	12.5
Finger bleeding	1	12.5

**Fig. 1:**
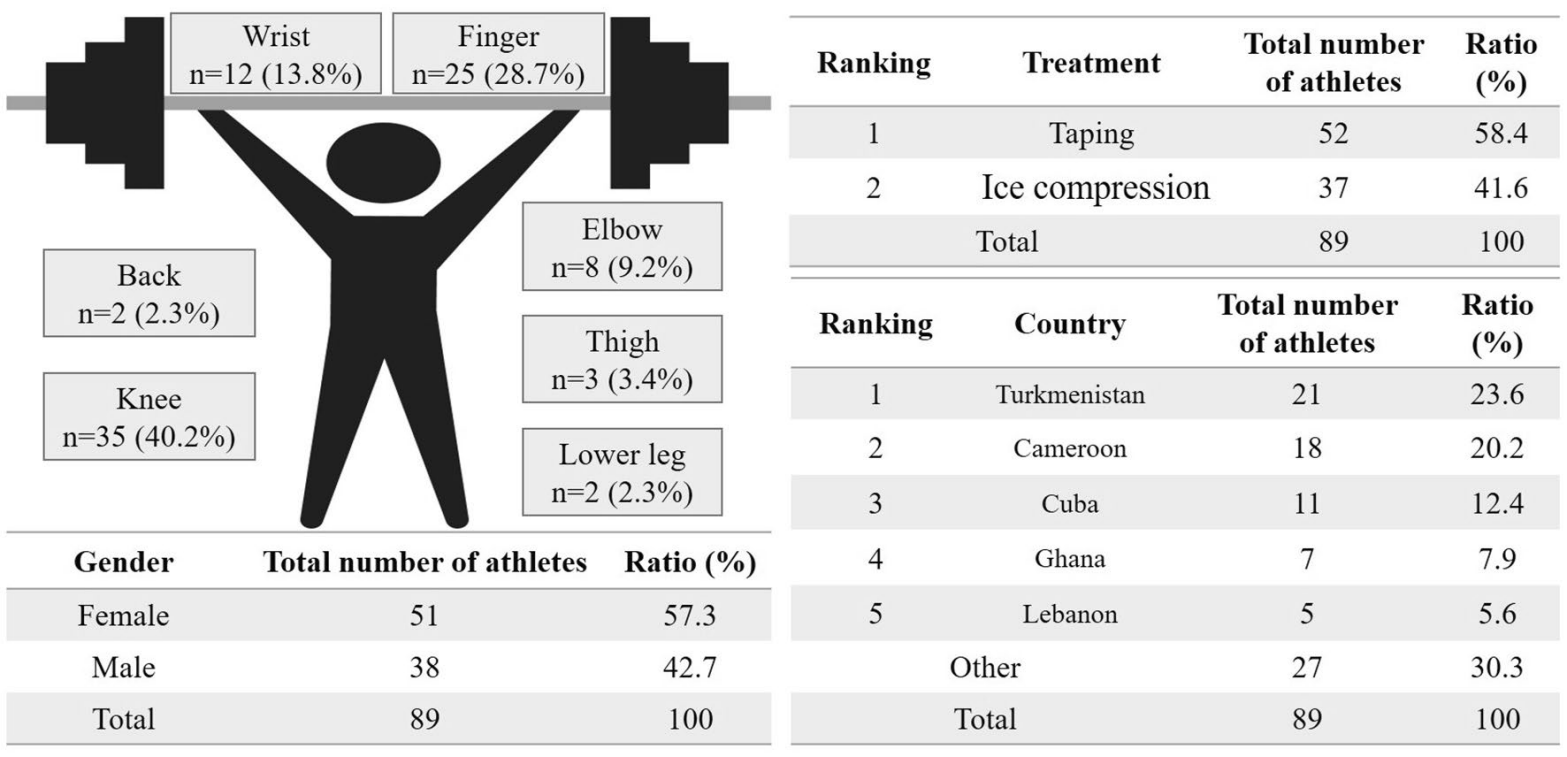
Medical procedures performed at the training floor of the weightlifting event of the Tokyo 2020 Olympic Games. A total of 89 patients required treatment. The body part with the highest number of treatments was the knee, followed by the finger, wrist, elbow, and thigh

At the first-aid station on the training floor, 89 medical procedures (51 performed on females) were performed (Fig. 1). The procedures comprised 52 tapings and 37 ice compressions. The most injured body part was the knee (*n* = 35, 40.2%), followed by the finger (*n* = 25, 28.7%), wrist *(n* = 12, 13.8%), elbow (*n* = 8, 9.2%), thigh (*n* = 3, 3.4%), and lower leg or back (*n* = 2, 2.3%). The country with the most athletes that underwent a medical procedure was Turkmenistan (*n* = 21, 23.6%), followed by Cameroon (*n* = 18, 20.2%), Cuba (*n* = 11, 12.4%), Ghana (*n* = 7, 7.9%), Lebanon (*n* = 5. 5.6%), and others (*n* = 27, 30.3%).

**Fig. 2:**
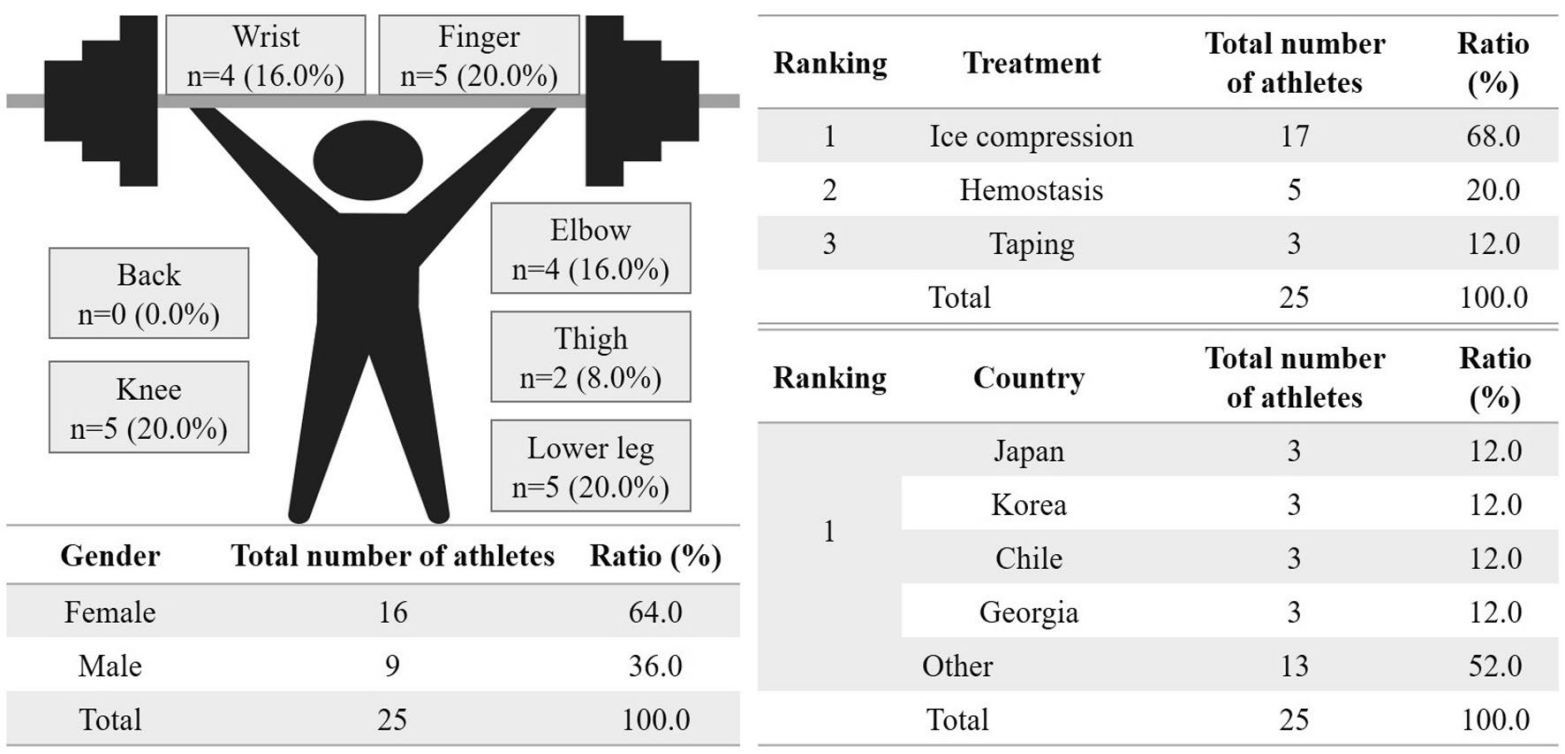
Medical procedures performed at the match floor of the weightlifting event of the Tokyo 2020 Olympic Games. A total of 25 patients needed treatment. The body parts with the highest number of treatments was the finger, knee, and lower leg, followed by the wrist and elbow

At the first-aid station on the match floor, there were 25 medical procedures, 16 of which were performed on females (Fig. 2). Of the procedures, 17 ice compressions, 5 haemostasis, and 3 tapings were performed. The body parts wherein procedures were most frequently performed are the finger, knee, and lower leg (*n* = 5, 20.0%), followed by the wrist and elbow (*n* = 4, 16.0%) and thigh (*n* = 2, 8.0%). The athletes who underwent a medical procedure in this station were mostly from Japan, Korea, Chile, and Georgia (*n* = 3, 12.0%), and the rest were from other countries (*n* = 13, 52.0%).

## Discussion

This study investigated the medical care provision in the weightlifting event venue of the Tokyo 2020 Olympic Games. In this study, the knees, fingers, and wrists were the most frequently treated areas in the first-aid station of the training floor, whereas the fingers, knees, and lower leg were most frequently treated in the first-aid station of the warm-up area. In contrast, for cases that required medical examination by a physician, most of the symptoms examined at the athlete medical station were medical or psychiatric. The symptoms encountered in the FOP area mostly involved the musculoskeletal system, except for two cases of light-headedness.

According to a previous report from the 2016 Rio de Janeiro Olympic Games, 34 (13.3%) weightlifting athletes were examined for injuries [1]. This frequency was higher than in other competitions. However, in the present study, a total of 17 athletes needed to be examined by a doctor. This difference is thought to be due to the fact that the previous report was the sum of the polyclinic at the Olympic village and the medical care at the venue, whereas this report was limited to medical care at the venue.

In a previous study, the elbows, shoulders, and hips were the main trauma sites. However, in the present study, none of the athletes required physical examination of the shoulder and hip. This may be because injuries in these areas are often of chronic course, and procedures that can be performed at the medical station are limited when it comes to these injuries.

Weightlifting is a sport with detrimental effects on the body; however, a previous report showed that the frequency of injuries of top-athlete states was equivalent to other non-contact sports [8]. Due to the nature of the sport, chronic symptoms are often observed [9], and these cases are not managed by medical stations at the competition venue. Additionally, that sudden injury may appear when an excessive load is applied in a special competition.

Regarding medical symptoms, there were few cases examined at the competition venue. However, previous reports have shown that all athletes had an illness at least once before the Olympic Games and had poor mental health [10]. Therefore, it should be noted that there are more athletes at risk than expected, both physically and mentally. Furthermore, an analysis of athletes who participated in the Olympic Games reported that the female sex, low energy availability, and poor mental health are risk factors for an injury. Thus, it is crucial to gain a comprehensive understanding of the medical examinations undergone by the athletes [11].

Additionally, in this study, it was found that there are many opportunities for medical professionals other than doctors to treat athletes. In preparation for the Olympic Games, this author attended the event after training on life-saving and haemostasic measures [12], but sometimes the inexperienced staff could not perform the treatment well. Therefore, it is very important to know in advance what kind of measures will be required at each competition venue.

### Limitations

The present study had a few limitations. This study only considered the medical care provided inside the weightlifting competition venue at the Tokyo 2020 Olympics Games and did not account for that provided outside the venue, such as in the polyclinic at the Olympic village. Therefore, in most cases, a diagnosis cannot be made. Additionally, it was not possible to investigate the athletes’ history and whether the chronic disease worsened or whether they sustained a new injury or illness. Finally, the medical service in this event was provided to the target population free of charge. This may affect the reception of medical services at the venue because there are differences in the quality of medical services in the countries from which the target population hailed.

## Conclusion

In the present study, majority of the athletes needed treatment; however, the injuries were not too severe and only a few needed medical care from a physician. This may be due to the nature of the sport of weightlifting and the peculiarity of the medical care in the stadium. We hope that this research will be useful in providing medical management in future major weightlifting competitions.

## Data Availability

The datasets generated during and/or analysed during the current study are not publicly available.

## Abbreviations

IF: International Federation
FOP: Field of play
IOC: International Olympic Committee
